# Loss of life years due to unavailable helicopter emergency medical service: a single base study from a rural area of Norway

**DOI:** 10.1080/02813432.2019.1608056

**Published:** 2019-04-29

**Authors:** Erik Zakariassen, Øyvind Østerås, Dag Ståle Nystøyl, Hans Johan Breidablik, Eivind Solheim, Guttorm Brattebø, Vegard S. Ellensen, Jana Midelfart Hoff, Knut Hordnes, Arne Aksnes, Jon-Kenneth Heltne, Steinar Hunskaar, Ragnar Hotvedt

**Affiliations:** a Department of Global Public Health and Primary Care, University of Bergen, Bergen, Norway;; b National Centre for Emergency Primary Health Care, Uni Research, Bergen, Norway;; c Department of Anaesthesia and Intensive Care, Haukeland University Hospital, Bergen, Norway;; d Department of Clinical Medicine, University of Bergen, Bergen, Norway;; e Department of Research, Norwegian Air Ambulance Foundation, Drøbak, Norway;; f Department of Research and Development, District General Hospital of Førde, Førde, Norway;; g Department of Heart Disease, Haukeland University Hospital, Bergen, Norway;; h Norwegian National Advisory Unit on Trauma, Division of Emergencies and Critical Care, Oslo University Hospital, Oslo, Norway;; i Section of Cardiothoracic Surgery, Department of Heart Disease, Haukeland University Hospital, Bergen, Norway;; j Department of Neurology, Haukeland University Hospital, Bergen, Norway;; k Center for Day Surgery, Hospitalet Betanien, Bergen, Norway;; l The Emergency and Primary Health Care Services, Kvam, Norway;; m Institute of Community Medicine, Faculty of Health Sciences, UiT The Arctic University of Norway, Tromsø, Norway

**Keywords:** Emergency medicine system, Primary health care, Air ambulance, rural area

## Abstract

**Background:** Despite the potential benefits of physician-staffed Helicopter Emergency Medical Service (HEMS), many dispatches to primary HEMS missions in Norway are cancelled before patient encounter. Information is sparse regarding the health consequences when medically indicated HEMS missions are cancelled and the patients are treated by a GP and ambulance staff only. We aimed to estimate the potential loss of life years for patients in these situations.

**Method:** We included all HEMS requests in the period 2010–2013 from Sogn and Fjordane County that were medically indicated but subsequently cancelled. This provided a selection of patients, with the purpose of studying cancellations independently of the patient’s medical status A multidisciplinary expert panel retrospectively assessed each patient’s potential loss of life years due to the lack of helicopter transport and intervention by a HEMS physician.

**Results:** The study included 184 patients from 176 missions. Because of unavailable HEMS, seven patients (4%) were anticipated to have lost a total of 18 life years. Three patients suffered from myocardial infarction, three from stroke and one from abdominal haemorrhage. The main contribution from HEMS care in these seven cases might have been rapid transport to definitive care. The probability of a patient losing life years when in need of HEMS evacuation was found to be 0.2%.

**Conclusion:** During the four years period seven patients lost 18 life years. Lack of rapid transport seems to be the primary cause of lost life years in this specific geographical area.

**Key Points**
- Knowledge about to what extent HEMS contributes to an increased survival and a better outcome for patients is limited.
- Compared to similar studies on life years gained the estimated loss of life years was minor when HEMS evacuation was unavailable in this rural area.
- The findings indicates that lack of rapid HEMS transport was the primary cause of the estimated loss of life years.

## Introduction

The challenges of providing emergency missions in rural areas are well known in both Norway and other countries [1–4]. Long distances and small hospitals with limited resources increase the need for Helicopter Emergency Medical Services (HEMS), but inclement weather conditions reduce HEMS’ availability. To what extent HEMS contributes to an increased survival and a better outcome for patients has been discussioned. The advantages of HEMS for trauma patients have been described in several studies [1,5–8]. However, a Cochrane review on the use of HEMS in adult trauma patients concluded that it is still unclear which elements provided by HEMS are beneficial for the patients [9]. Two studies from Norway have concluded that life years were gained [10,11]. Observational study designs are most common, which limits the validity and generalization of the study results. Randomization to mode of transportation in emergency cases has both ethical and practical concerns.

In Norway, the general practitioner (GP) on-call is an important contributor in emergency medicine, together with the ambulance service [12–13]. HEMS is an integrated part of the emergency medical system and is to be used for cases of illness or injuries that require rapid transport, clinical assessment, or advanced treatment.

Sogn and Fjordane county (SF county) is a rural part of Western Norway. Of all HEMS requests in 2014, 40% were cancelled. Figures from the National Air Ambulance Service showed that the most common cause for not completing a mission was stated as “no longer medical indication” (30%), followed by “bad weather conditions” (6%). Technical problems, exceeded duty time for the crewmembers, or concurrent missions were less frequent (4%) [14].

The health consequences of unavailable HEMS, in cases where advanced life support or rapid transport is deemed necessary, are unknown. This is relevant when discussions regarding centralization of ambulances and GPs out-of-hour service in the county. The aim of our study was to estimate the potential loss of life years when medically indicated missions were cancelled.

## Methods

### Setting and data sources

SF county consists of 26 sparsely populated municipalities with a total of 108,000 inhabitants. It spans 200 kilometres west to east and 130 kilometres south to north. The challenging geography with mountains, fjords, islands, and poor roads quality increases response time for ground ambulances. Especially during winter, weather conditions with reduced visibility are common. There are 15 out-of-hours emergency services in the area, each with one general practitioner (GP) on call. A total of 21 ground ambulance stations are localized throughout the county.

**Figure 1.**
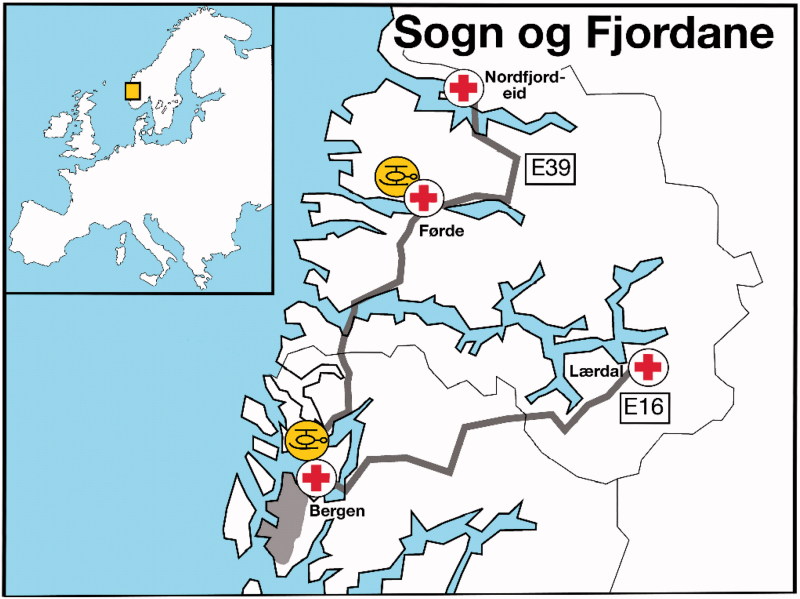
Map of the geographical area with hospitals, roads and HEMS bases. Link to map of HEMS bases in Norway with 30 minutes flying time circles. http://www.luftambulanse.no/sites/default/files/LAT-kart-2015.pdf

There are three hospitals in the county; Førde, Nordfjordeid and Lærdal (Figure 1). The latter two provide services for medical emergencies only. However, there is always an anaesthesiologist on call in all hospitals. The main hospital in Førde has emergency services for most common medical and surgical/(incl. trauma) conditions. Patients with major trauma, severe burns, a need of percutaneous coronary intervention (PCI), or with other severe medical conditions are transported after emergency treatment (or directly from scene) to Haukeland University Hospital in Bergen. Transport time by ground ambulance from Nordfjordeid and Lærdal to Førde is 90 and 120 minutes, respectively. From Lærdal and Førde to Bergen, 150 and 130 minutes, respectively. A ferry crossing is necessary for all routes, except from Lærdal to Bergen. The Emergency Medical Dispatch Centre (EMCC) is located at Førde.

One HEMS is located in Førde. The team consists of a pilot, an anaesthesiologist, and a rescue paramedic. It covers most of the county within a 20 minute one-way flight time. HEMS in neighbouring counties also perform missions in SF county when needed. Additionally, the military operates an anaesthesiologist-staffed Search and Rescue (SAR) helicopter located in Florø, which also responds to emergency medical missions, if needed. All HEMS bases in Norway have a rapid response car available. If weather conditions restrict flight, it is an option to transport the anaesthesiologist to the patient by car.

HEMS requests are registered in the AirDoc activity registration database, which was used for identification and inclusion of missions, and to identify the patients in the Acute Medical Information System (AMIS), in which all alarm calls to EMCC are registered. AMIS contains patient information and administrative response data (including date, time of dispatch of prehospital resources, responding unit, response time, and where the patient was transported).

All HEMS requests in SF county for the years 2010–2013 were identified. SAR data were available from 2012. Both primary missions (on-scene missions) and secondary missions (inter-hospital transports) were included.

Due to cancellation of HEMS the included patients were transported to hospital by ground ambulance. Ambulance personnel and GPs on call provided treatment. Subsequently the physicians at local hospitals also treated the patients. Patient records from GPs, ground ambulances and the hospitals were collected and made available for assessment. Symptom categories were based on the clinical information available during HEMS dispatch.

### Case definition and study design

A cancellation was defined as either a declined mission before helicopter take-off or an aborted mission after take-off. Only medically-indicated missions that were subsequently cancelled due to non-medical reasons were included. Missions performed with a rapid response car were also excluded. This provided a selection of patients with the purpose of studying cancellations independently of the patient’s medical status. This observational study was thus designed to include a case mix not biased by patient-related clinical information as a reason for cancellation.

### Case assessments

Written case reports were prepared for each of the included patients by one of the authors (DSN), based on medical records from the prehospital services and discharge summary from hospitals, including symptoms, clinical signs, other known diseases (comorbidity), preliminary International Classification of Primary Care (ICPC-2) and International Classification of Diseases (ICD10) at discharge from hospital. Data on medical interventions, time intervals, approximate transportation time to desired hospital if the HEMS had arrived, and hospital stay were recorded and assessed. Direct flight track times were calculated based on information from the National Air Ambulance Services, and the approximate transportation time from the location to the relevant hospital was calculated. The remaining life expectancy based on Norwegian life expectancy tables was found (Statistics Norway) for each patient [15].

An anaesthesiologist from a different HEMS unit then assessed the case reports. Alternative treatment(s) was described in addition to the potential destination hospital if an HEMS evacuation had taken place.

### Loss of life years estimations

A multidisciplinary expert panel assessed the patients’ potential loss of life years (nominal group process) exclusively due to the lack of helicopter transport and potential interventions by an anaesthesiologist [16]. The panel consisted of an anaesthesiologist (GB), a cardiologist (ES), a general practitioner (AA), a neurologist (JMH), an obstetrician (KH) and a surgeon (VSE). Due to broad experience in emergency medicine both pre- and in-hospital, three of the members (GB, ES, AA) received all the case reports, while the others (JMH, KH, VSE) received reports within their specific area of expertise. Loss of life years was estimated using the following algorithm:

1. The experts individually divided the cases into two groups, one with no anticipated loss of life years, the other with a potential loss of life years. Cases selected to the group “no anticipated loss of life years” by all the experts, were not further assessed.
2. Then, all experts assessed the case reports from the group of patients with potential loss of life years. Comorbidity at the time of incident, as well as the actual incident were used for adjusting expected remaining life years by the experts’ best estimates, and in accordance with literature [17–19]. For each patient, loss of life years was calculated as the difference between expected remaining life years after actual evacuation and the experts’ estimate of remaining life years if a HEMS evacuation had been available. The expert group assessed the following factors: transport mode, treatment performed, confirmed diagnosis at hospital discharge, and patient outcome. An example of potential life years lost could be a case of myocardial infarction with ST-segment elevation in ECG. This condition can be treated with thrombolysis or PCI, but unavailable helicopter transport increased the actual transport time to a hospital with PCI capability to more than 90 minutes.
3. The estimates and the experts’ arguments were presented at an expert group meeting. All steps from the individual assessment described above, were discussed thoroughly within the group with consensus on estimated loss of life years as a goal. In the event of disagreement, the mean of the various experts estimates of life years lost were used in the analysis.

### Statistical analysis and ethical approvals

Standard descriptive data analyses were performed. Age and expected remaining life years were presented as the median and interquartile range (IQR). Pearson Chi-Square tests were used to analyse differences between the two groups “possible life years lost” and “no life years lost”. A p-value of 0.05 or below was considered statistically significant. Data were entered and analysed using SPSS Statistics Version 22 (IBM Corp., Armonk, NY, USA). The probability of not getting medically indicated HEMS evacuation was calculated as the total number of declined and aborted missions divided by the total number of missions during the study period. Probability of loss of life years was calculated as number of patients with loss of life years divided by total number of patients.

The study was approved by the Regional Committee for Medical and Health Research Ethics (2013/373 REC West, Norway). All patient data were anonymized before assessment by the expert panel.

## Results

### Missions and patients

**Figure 2.**
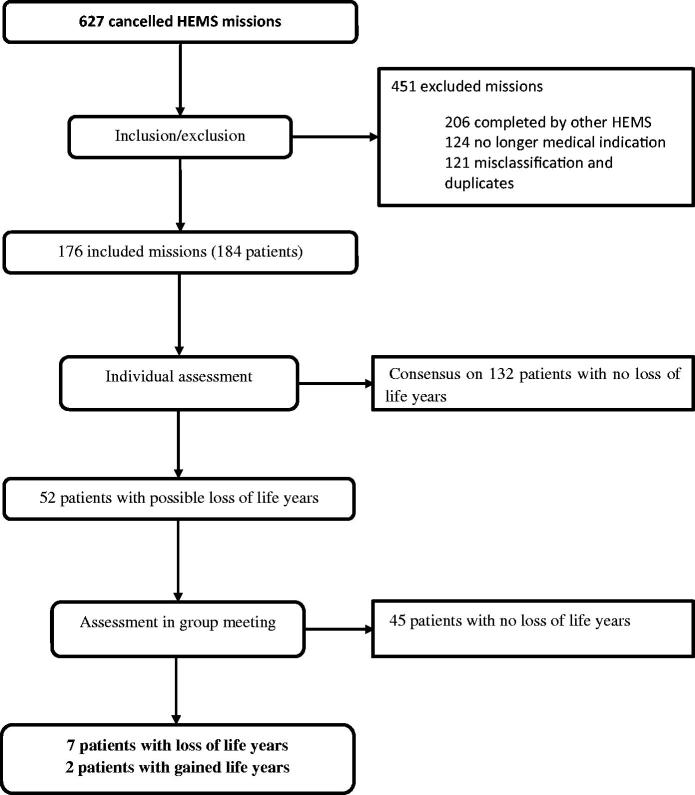
Flowchart showing included missions and patients.

During the study period, the total number of completed missions was 2,582 for HEMS Førde and SAR Florø combined. There were 627 cancelled missions (24%; Figure 2). However, the majority of these missions (72%) were excluded; 33% were completed by another HEMS, 20% were cancelled due to “no longer medical indication”, and 19% were excluded due to duplicates. The 176 remaining cancelled missions involved 184 patients. The probability of not getting a medically indicated HEMS evacuation in SF County during the study period was thus 5.9%.

The median age of the 184 patients was 59 years (IQR 31-72), and 61% were male. Median remaining expected life years was 25 years (IQR 15-52). Cardiac and neurologic diseases were the two most frequent medical conditions (35% and 20%, respectively), while trauma patients constituted 14%. The proportion of patients with cardiology conditions was higher in the group of “possible life years lost”, compared to the group “no life years lost”. For trauma patients, the opposite was found (Table 1).

**Table 1.** Demographic data on included patients. In the first assessment the patients (*n* = 184) were divided by the experts between the groups “possible life years lost” and “no life years lost”; gender, mission type, patient’s location and type of patient when HEMS was alerted.

	Possible life years lost^a^ (*N* = 52)		No life years lost (*N* = 132)		
Variables	*n*	(%)	*n*	(%)	*P* value
Gender					0.83
Female	21	(40)	51	(39)	
Type of mission					0.87
Primary mission	34	(65)	88	(67)	
Location					0.52
Home	23	(44)	54	(41)	
Primary health care	4	(8)	11	(8)	
Public place	8	(16)	24	(18)	
Hospital	17	(32)	43	(33)	
Type of patient					0.01
Cardiology	27	(52)	38	(29)	
Neurology	14	(26)	22	(17)	
Trauma	3	(6)	24	(18)	
Infection	3	(6)	10	(8)	
Surgery	3	(6)	7	(5)	
Obstetrics	1	(2)	11	(8)	
Other	1	(2)	10	(8)	
Breathing difficulties	0		7	(5)	
Intoxication	0		3	(2)	

Pearson Chi-Square tests were used to analyse for statistically significant differences between the groups.

^a^Possible life years lost after first round of classification.

### Loss of life years

During the first selection stage, 52 (28%) of the 184 patients were identified with a potential loss of life years. The expert panel finally concluded that 7 of these (4%) had most likely lost a total of 18 life years (Table 2). Consensus on estimated loss of life years was achieved in all patients. The probability of a patient losing life years when in need of HEMS evacuation was found to be 0.2%. Three of the seven patients were transported from local hospitals to a higher level of care. Two patients were found to have gained life years (Table 2).

**Table 2.** Patients with an estimated loss of life years, main emergency medical condition, reasons for estimated loss of life years and mission type for 9 missions.

Estimated loss of life	Main emergency medical		
years	condition (ICD-10)	Reason for loss of life years	Mission type
**9.5**	Abdominal haemorrhage (I72.8)	Survived to hospital admission (Førde) from local hospital, delayed by 1h 40 min than if transported by helicopter. Surgical procedures were available, but the patient suffered circulatory collapse and died of haemorrhage in the ER at Førde hospital. Autopsy demonstrated a ruptured, dissecting aneurysm in a. mesenterica sup.	Secondary
**5.0**	Cerebral infarction (I63.3)	Delayed start of thrombolytic treatment. Sequelae; hemiparesis, aphasia and apraxia.	Primary
**2.0**	Cerebral haemorrhage (I61.8)	The patient did not reach PCI centre and suffered a cerebral haemorrhage as side effect of thrombolytic treatment. Sequelae (after evacuation of hematoma): hemiparesis.	Secondary
**1.0**	Cerebral infarction (I63.9)	Delayed start of thrombolytic treatment and lack of facilities for thrombectomy. Sequelae: hemiparesis, facial paralysis.	Primary
**0.3**	Myocardial infarction (I21.4)	The patient arrived at local hospital 2 hours after estimated air transport arrival to PCI centre, too late for thrombolytic treatment. He received conservative treatment only. If transported to HUS, revascularization within 3-4 hours after debut of symptoms would have been possible, reducing infarction size and improving life expectancy. Sequelae: major damage apically with akinesia and thin-walled myocardium.	Primary
**0.2**	Myocardial infarction (I21.1)	Revascularization delayed by 1h 30m. Earlier treatment would have reduced the infarction size, and the transport delay influenced life expectancy. Sequelae: concentric hypertrophy and anterolateral hypokinesia.	Primary
**0.2**	Myocardial infarction (I21.0)	The time from debut of symptoms was >6 h at arrival, with ST elevations still present. There was still indication for acute PCI, but not for thrombolytic treatment. The abortion of air transport resulted in conservative treatment; revascularization was performed 6 days later. An acute PCI could have decreased infarction size and improved life expectancy. Sequelae: anterolateral hypokinesia	Secondary
**−0.1** **−0.2**	Myocardial infarction (I21.0)	Both patients with gained life years received thrombolytic treatment with documented good clinical outcome (pain relief, normalization of ECG and flow in the actual artery at the following coronary angiography) within a shorter time than possibly obtained by revascularization after helicopter transport to the PCI centre.	Primary

All patients were adults (47–80 years).

ICD-10 is an international classification of diseases retrieved from hospital records of the patients. Mission type; Primary mission is response to a patient outside hospital and secondary mission is inter-hospital transport.

Median age for the seven patients with loss of life years and for the two patients with life years gained was 69 years (IQR 58-77), and median adjusted life expectancy was 10 years (IQR 4-11). Colorectal cancer, prostate cancer, atrial fibrillation, stroke, depression, hypercholesterolaemia, COPD, and a history of smoking were the main causes of reduced life expectancy. The total remaining life years for the seven patients with loss of life years was estimated to 158 years before adjustments, and 83 years when adjusted for comorbidity.

In most cases, lack of rapid transport to the hospital in Bergen was considered to be the main cause of loss of life years, rather than lack of advanced treatment. One patient who died may have been saved at the hospital in Førde, but arrived too late. Five other patients died, at site of cardiac arrest. Ambulance crew and GPs on call started and terminated CPR at site.

## Discussion

The expert panel concluded that loss of life years due to unavailable HEMS evacuation was minor in this specific rural area of Norway. A large part of missions that were cancelled by Førde HEMS due to weather conditions or other non-medical reasons was handled by neighbouring HEMS units. Hence, the probability of a patient experiencing a lack of HEMS, and then subsequently experiencing a loss of life years was very low. This is one of the positive effects of the high density of HEMS bases in Western Norway [20] and active GPs together with the ground ambulance service.

### Strengths and limitations

Our study has important strengths; all relevant mission and patient data were available and retrieved; the expert panel consisted of persons with no affiliations to HEMS Førde or the health authorities in SF county; the panel reached a consensus in all cases; and the method (a nominal group process) has been used in similar studies [10,11,15,21,22].

An expert panel consisting of six different persons will not conclude with exactly the same estimates. Nonetheless, the reliability of the method is acknowledged [21]. All cases are from a single rural HEMS base. The prehospital emergency system in Norway is well developed, and GPs on call and local hospitals are capable of giving advanced treatment, like thrombolysis. This may reduce the external validity of the findings to services in other countries and the presented results must be interpreted with caution.

Weather or other non-medical reasons were anticipated to be the main reason for declined or aborted HEMS missions, unrelated to the patient’s clinical condition. When assessing the distribution of diagnoses we found that trauma patients constituted 14% in our study, in contrast to approximately 30% for HEMS missions on the west coast and for Norway as a whole [15,23]. In addition, both cerebral infarction and myocardial infarction were the main problem among the patients that lost life years. This may indicate a lower response threshold for the HEMS crew in trauma missions. The decision to undertake a flight in bad weather is the pilot’s decision, but may be influenced by the patient’s condition and the total experience of the crew.

Measure of loss or gained life years can be interpreted as a narrow measure of HEMS utility. Other utility measures could e.g. be sequelae after stroke, quality of life score among stroke patients and length of hospitalisation.

Our chosen method may have led to a selection bias. That such a large share of missions was handled by neighbouring HEMS units was unexpected, and resulted in a much smaller number of included missions than our preliminary calculations. There were no children or young adults with estimated loss of life years in this study. In studies where life years gained are estimated, children have a major impact on the results [10,11]. Missions using the rapid response car for transport to patients were also excluded. Hence, some seriously ill or injured patients may not have been included due to treatment by crew from a neighbouring HEMS unit and/or the use of rapid response car. This increases the uncertainty of the calculated loss of life years, as one or a few patients could have a major impact on the results. This was confirmed by the fact that one patient in our material represented more than half of the total loss of life years. If this patient was an outlier, the mean loss from the rest of the patients was barely clinically significant.

### Comparison with previous studies

The expert panel concluded loss of life years for 4% of the patients. One Norwegian study found that life years were gained by 7% of the patients attended by HEMS, with an average of 6.8 years per patient [11]. Another study from Norway concluded that 89% of the patients transported by a physician-staffed HEMS would have done just as well in a ground ambulance without a physician [10]. A new publication showed no differences in survival to discharge between patients taken care of by HEMS, compared to the group of patients not taken care of by HEMS due to concurrencies [24]. This indicates a low threshold for using HEMS (possible overtriage), if lifesaving treatment is the main goal of HEMS. Delgado et al. have also discussed presence of overtriage in a cost-benefit context of helicopter use; less transport of minor injuries will improve cost-effectiveness [25]. In 2011, HEMS Førde had a three times higher rate of missions per inhabitant compared to the other HEMS bases in Norway [17]. Thus, overtriage may be an important contributing factor explaining the low proportion of patients with life years lost in our study. There is a lack of a national HEMS dispatch criteria, which could reduce overtriage. However, undertriage could have a very negative impact on patients’ outcome. We have to accept some degree of overtriage to avoid undertriage. Still, an important strength of HEMS in Norway is its flexibility. HEMS crew decision to accept a mission is based on several aspects like condition of the patient and patient’s distances to ambulance, GP and hospital.

Local GPs and ambulance personnel provide important treatments [26]. In such conditions, rapid transport might have been the main advantage of HEMS rather than advanced interventions. Contrary, in the case of abdominal haemorrhage, treatment with available blood products (erythrocytes and plasma) was started at the local hospital.

The experts concluded that two patients experienced a health benefit due to the lack of HEMS. These patients would have been transported to acute coronary intervention if HEMS was available. In both cases the patients received thrombolytic treatment with a documented good clinical outcome (pain relief, normalization of ECG and flow in the actual artery at the following coronary angiography) within a shorter time than potentially achieved by revascularization after helicopter transport to PCI centre. Reducing the myocardial ischaemia time period most probably reduced the infarction size and improved the life expectancy in these patients [27].

For the seven patients with loss of life years, the HEMS physician chose not to use the rapid response car. Hence, unavailable rapid transport to advanced treatment in hospitals seems to be the main factor for loss of life years. Another study on the same patients indicated that in cases when HEMS units were not available, ambulance personnel, GPs and physicians at local hospitals provided appropriate emergency procedures and treatments [26]. A study based on data from Hotvedt et al. 1996, concluded that Norwegian GPs could provide adequate treatment to more than half of the patients treated by an HEMS doctor [28]. Another study on HEMS patients from the northern part of Norway concluded that GPs often started important medical treatment, if needed, before HEMS arrival [29].

## Conclusion

During the four years period seven patients lost 18 life years. The findings indicates that lack of rapid HEMS transport was the primary cause of the estimated loss of life years.
